# Clear cell urothelial carcinoma of the urinary bladder: a case report and review of the literature

**DOI:** 10.1186/1752-1947-8-275

**Published:** 2014-08-14

**Authors:** Virginia M Knez, Willis Barrow, M Scott Lucia, Shandra Wilson, Francisco G La Rosa

**Affiliations:** 1 Department of Pathology, School of Medicine, University of Colorado, Anschutz Medical Campus, Aurora, CO 80045, USA; 2 Department of Urologic Oncology, School of Medicine, University of Colorado, Anschutz Medical Campus, Aurora, CO 80045, USA

**Keywords:** Clear cell, Cancer, Urinary bladder, Urothelial carcinoma

## Abstract

**Introduction:**

The occurrence of clear cell tumors in the bladder is not uncommon. Clear cell dysplasia is well-described and characterized by focal replacement of transitional mucosa by cells with abundant clear cytoplasm, nuclear enlargement, and a granular chromatin pattern. Clear cells can also be seen in clear cell adenocarcinoma, which is rare, comprising 0.5% to 2.0% of the reported bladder carcinomas. Other clear cell tumors found in the bladder to be considered in the differential diagnosis are tumors of Müllerian origin and metastatic lesions, such as renal cell carcinoma, clear cell sarcoma, and malignant melanoma. Clear cell urothelial carcinoma is exceedingly rare, with only nine clinical cases described in the literature.

**Case presentation:**

We report the case of a 75-year-old Caucasian man who presented with intermittent hematuria, in whom a bladder tumor was identified. A final histopathology examination of a cystoprostatectomy specimen revealed a pT3b, G3 urothelial carcinoma of clear cell type (>90% clear cells) and a prostatic adenocarcinoma of Gleason grade 3+3 (score=6). The bladder tumor consisted of sheets of malignant cells with severe nuclear atypia and abundant clear cytoplasm; no glandular or tubular structures were identified. Tumor cells were periodic acid-Schiff positive and negative after diastase treatment; additional mucicarmine and oil red O stains were negative. Immunohistochemical stains showed the tumor cells positive for cytokeratin 7 (CK7), p63 (>80% nuclei), p53 (about 30% nuclei), vimentin, E-cadherin, cluster of differentiation (CD10), and Ki-67 (>70% nuclei). Stains for cell adhesion molecule 5.2 (CAM 5.2), CD117, cytokeratin 20 (CK20), human melanoma black 45 (HMB-45), paired box protein (PAX 8), placental alkaline phosphatase (PLAP), prostate specific antigen (PSA), renal cell carcinoma (RCC), cancer antigen 25 (CA25), leukocyte common antigen (LC), S-100 protein, and uroplakin III were all negative.

**Conclusions:**

The tumor marker profile was consistent with clear cell type carcinoma of urothelial origin. Within the differential diagnoses, we ruled out other possible tumor types such as urothelial carcinoma with focal clear cell differentiation, clear cell adenocarcinoma, Müllerian tumors, and metastatic disease.

## Introduction

Urinary bladder carcinomas that differ on histology from typical urothelial carcinomas were reviewed by Young and Eble [1]. These tumors account for approximately 15% of all bladder carcinomas and fall into four major categories: variant forms of urothelial carcinoma, squamous cell carcinoma, adenocarcinoma, and undifferentiated carcinoma [1]. Variant forms of urothelial carcinoma include squamous and glandular differentiation, but these are only focal changes, with typical urothelial carcinoma seen elsewhere in the tumor [1].

The identification of clear cell tumors in the bladder is not uncommon [2]. Clear cell dysplasia is well-described and characterized by focal replacement of transitional mucosa by cells with abundant clear cytoplasm, nuclear enlargement, and a granular chromatin pattern [3]. Clear cells can also be seen in clear cell adenocarcinoma, which is rare, comprising 0.5% to 2.0% of the reported bladder carcinomas [1]. The name clear cell adenocarcinoma is used because of the histologic similarity to clear cell adenocarcinomas of the female genital tract [1]. Clear cell urothelial carcinoma is exceedingly rare, with only nine clinical cases described in the literature [2,4-7]. Other clear cell tumors found in the bladder to be considered in the differential diagnosis are metastatic lesions, such as renal cell carcinoma or melanoma.

We report the case of a male patient who underwent radical cystoprostatectomy for a urinary bladder tumor featuring rounded to polygonal cells with abundant clear cytoplasm, which deeply infiltrated the vesical wall. We discuss the morphologic features, immunohistochemical, and special staining pattern, and review the literature on clear cell urothelial carcinoma.

## Case presentation

Our patient was a 75-year-old Caucasian man with a history of intermittent hematuria for a few days the month preceding a cystoscopy. He was otherwise asymptomatic and denied bone pain, jaundice, hemoptysis, or weight loss. Of clinical relevance, he was a former smoker who quit 21 years ago and had been using smokeless tobacco for the last 16 years. His referring institution performed a cystoscopy revealing a bladder tumor and a biopsy that showed a high-grade urothelial carcinoma with widespread clear cell differentiation (>80%), with severe necrosis, and extensive invasion into the muscularis propria. An ultrasound at the time showed a large urinary bladder mass with no evidence of extravesical disease, and mild right-sided hydronephrosis suggestive of obstruction of his distal right ureter. Because of an elevated serum creatinine of 1.6mg/dL, neoadjuvant chemotherapy was not attempted. A radical cystoprostatectomy was soon performed at our institution. On follow-up, his creatinine levels continued to be elevated and cisplatinum-based chemotherapy was further deferred. After one year of follow-up, our patient had no evidence of disease recurrence.

**Figure 1:**
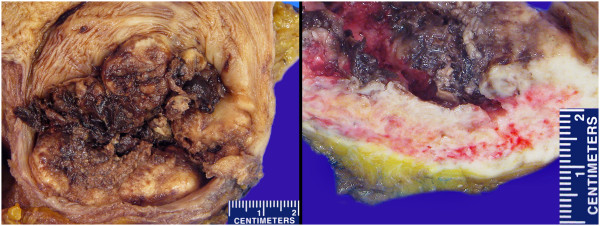
**Macroscopic examination of the cystectomy specimen.** Gross examination showing a fungated hemorrhagic mass measuring 5.5×5.0×2.3cm, involving the inferior right lateral wall, and with near obliteration of the right ureteral orifice (left). Cut section of the tumor shows full thickness invasion through the detrusor muscle and into the perivesical adipose tissue (right).

**Figure 2:**
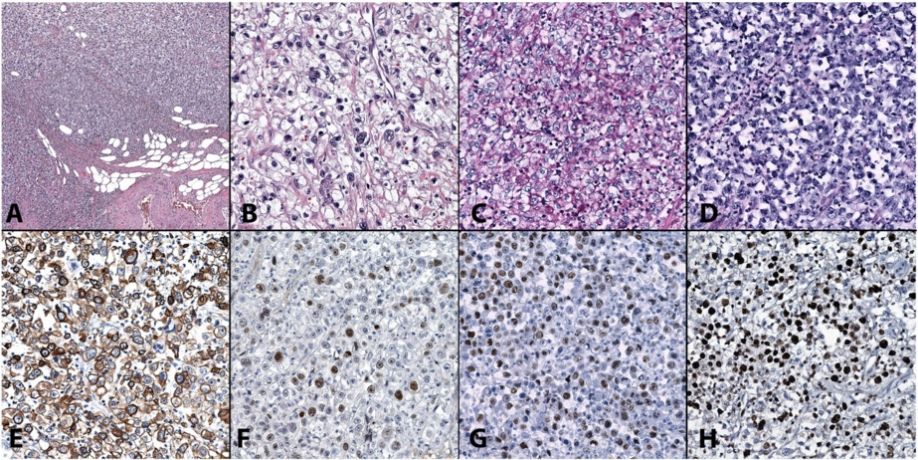
**Hematoxylin and eosin staining of the cystectomy specimen. (A)** Sheets of clear cells can be seen invading through the muscularis propria into the perivesical adipose tissue (10× objective). **(B)** Rounded to polygonal malignant cells with severe nuclear atypia, prominent nucleoli, and abundant, clear cytoplasm seen under a 40× objective. **(C)** These cells were positive for periodic acid-Schiff, which **(D)** disappeared with diastase treatment. **(E-H)** Immunoperoxidase stains show tumor cells with strong positive membrane staining for CK7 **(E)**, approximately 30% nuclear staining for p53 antigen **(F)**, >80% nuclear staining for p63 antigen **(G)**, and >70% nuclear staining for Ki-67 (MIB-1) antigen **(H)**. Figures B through H at 40× objective.

The cystoprostatectomy specimen was fixed in 10% buffered formalin, and the prostate was dissected out for independent processing. Macroscopic examination showed a fungated and hemorrhagic urinary bladder mass arising from the mucosal surface, measuring 5.5×5.0×2.3cm, involving the inferior right lateral wall, and with near obliteration of the right ureteral orifice (Figure 1). Representative sections of the bladder tumor were embedded in paraffin, sectioned at 5μm, and stained with hematoxylin-eosin. Microscopic examination revealed a tumor consisting of sheets and nodules of rounded to polygonal malignant cells with enlarged nuclei, prominent nucleoli, and abundant clear cytoplasm (>90% clear cell differentiation) (Figure 2A,B). No glandular differentiation or hobnail cells were observed. The tumor invaded through the full thickness of the muscularis propria (detrusor muscle) into the perivesical adipose tissue, approaching within 0.5mm of the perivesical serosal surface (pathology stage pT3b). Extensive necrosis accompanied by prominent acute inflammation was present. Lymphovascular invasion was identified. Focal carcinoma *in situ* was present in the adjacent surface urothelium. This tumor showed very similar histopathology to the prior bladder biopsy from the referring institution.

The prostate gland revealed multifocal and bilateral prostatic adenocarcinoma, Gleason grade 3+3 (score=6), measuring 6mm in greatest dimension and involving <5% of the prostate. There was focal extension of prostatic adenocarcinoma into the left posterior surgical margin (2mm in length).To characterize the content of the clear, vacuolated cytoplasm of the malignant cells in the bladder tumor, special histochemical stains were performed. The tumor cells were positive for periodic acid-Schiff (PAS) staining, which disappeared with diastase (PAS-D) treatment (Figure 2C,D). The cells were negative for mucicarmine staining and for oil red O on frozen sections of the formalin-fixed tissue. This staining pattern confirmed the cells contained polysaccharides such as glycogen, as opposed to mucin or fat.

Immunohistochemical stains were done with the following markers: Cytokeratin (CK) 7, CK20, Cell adhesion molecule (CAM) 5.2, p63, Uroplakin III, Vimentin, E-cadherin, p53, Cluster of differentiation (CD) 10, Paired box protein (PAX) 8, Renal cell carcinoma (RCC), CD117, Placental alkaline phosphatase (PLAP), S-100 protein, Human melanoma black (HMB) 45, Prostate Specific Antigen (PSA), Cancer Antigen 125 (CA-125), and Ki-67. Appropriate positive and negative controls were used. The tumor cells were positive for CK7 (Figure 2E), p53 (approximately 30% nuclear, Figure 2F), p63 (>80% nuclear, Figure 2G), Vimentin, E-cadherin (focal), CD10 (patchy), and Ki-67 (>70% nuclear, Figure 2H). The tumor cells were negative for Cancer antigen 125, CAM 5.2, CD117, CK20, HMB-45, PAX 8, PLAP, Prostate specific antigen, S-100 protein, Uroplakin III, and RCC. Additionally, the cells were negative for Leukocyte common antigen, a test previously performed and reported by the referring institution. This tumor marker profile ruled out clear cell carcinoma of Müllerian origin and other tissue origins and was most diagnostic of a primary urothelial origin. The immunostain and histochemical stain results are detailed in Table 1.

**Table 1:** Special immunoperoxidase and histochemical stains

**Antibody specificity**	**Tumor cell reactivity**	**Source**	**Clone**	**Positive controls**
Cytokeratin 7	Approx. 75%, cell membrane and cytoplasm	Dako	OV-TL12/30	Breast carcinoma
p53	Approx. 30%, nuclear	Dako	DO-7	Breast cancer
p63	>80%, nuclear	Biocare	4A4	Breast myoepithelial cells
Vimentin	Approx. 80%, cell membrane and cytoplasm	Ventana	3B4	Salivary gland
Ki-67	>70%, nuclear	Ventana	30-9	Tonsil
CD10	Approx. 15%, cell membrane	Cell Marque	56C6	Tonsil
E-cadherin	Approx. 10%, cell membrane	Cell Marque	ECH-6	Breast ductal carcinoma
CA125	Negative	Cell Marque	OC1-25	Ovarian carcinoma
CAM 5.2	Negative	Becton Dickinson	CAM 5.2	Cholangiocarcinoma
CD117	Negative	Dako	c-kit	Gastrointestinal stromal
Cytokeratin 20	Negative	Biocare	Ks 20.8	Large intestinal epithelium
MAA	Negative	Ventana	HMB-45	Melanoma
PAX 8	Negative	Cell Marque	MRQ-50	Nonmucinous ovarian
PLAP	Negative	Ventana	NB10	Placenta
PSA	Negative	Ventana	Rabbit polyc.	Prostate
RCC	Negative	Cell Marque	PN-15	Renal cell tubular epithelium
S-100 protein	Negative	Ventana	4C4.9	Melanoma
Uroplakin III	Negative	Cell Marque	SP73	Urothelial carcinoma
**Special histochemical stain**	**Reactivity**			
PAS	Positive cytoplasm	Ventana		Cirrhotic liver
PAS-diastase	Negative	Ventana		Cirrhotic liver
Mucicarmine	Negative	Manual		Small intestinal epithelium
Oil red O (frozen section)	Negative	Manual		Perivesical adipose tissue

CA, Cancer antigen; CAM, Cell adhesion molecule; CD, Cluster of differentiation; MAA, melanoma-associated antigen; PAS, periodic acid-Schiff; PAX, Paired box protein; PLAP, Placental alkaline phosphatase; PSA, Prostate-specific antigen; RCC, Renal cell carcinoma.

## Discussion

Urothelial carcinoma is the most common tumor arising in the urinary tract [4], and the most common variants have glandular or squamous differentiation [1]. Little is known, however, about urothelial carcinoma with predominantly clear cells, other than the few clinical cases reported previously, as listed in Table 2[5].

**Table 2:** Bibliographic references of case reports of clear cell urothelial carcinoma

**Patient**	**Clinical presentation**	**Pathology findings**	**Selected testing results**	**Additional history**	**Follow-up**	**Report**
71 M	Painless hematuria	Left wall involvement into perivesical fat	Glycogen positive	Prostatic adenocarcinoma	Death after 20 months	Kotliar *et al.*[6]
			Mucin negative	Gleason grade 2+3 (>25%)		1995
			PSA negative			
			PSAP negative			
			EM: no gland formation			
58 F	Dysuria and infected urethral cyst with pyuria	Urethral involvement with invasion	Glycogen positive		Data not available	Kotliar *et al.*[6]
						1995
			Mucin negative			
70 F	Intermittent, gross hematuria	Right upper ureter stenosing lesion	Glycogen positive		Alive at six months	Braslis *et al.*[4]
			Mucin negative			1997
70 M	Frequency, urgency, anuria	Red, irregular mucosa Invasion within 0.5mm of detrusor muscle.			Data not available	Braslis *et al.*[4]
						1997
70 M	Asymptomatic hematuria	Left wall tumor, muscle invasive, treated with TURBt		History of clear cell renal cell carcinoma, pulmonary metastasis	No recurrence after	Yamashita *et al.*[7]
						2006
					seven months	
69 F	Gross hematuria	Right wall tumor, treated with TURBt		Chronic renal failure, hemodialysis treatment	No recurrence after	Isono *et al.*[13]
						2010
					20 months	
82 M	Asymptomatic	Deep infiltration of muscularis propria	GATA3+	History of clear cell renal cell carcinoma,	Alive at 12 months	Rotellini *et al.*[2]
			UroVysion fluorescence *in situ* hybridization			
				Furhman grade 2		
						2010
67 M	Progressive lower urinary tract symptoms	Bilateral ureteral stenosis due to muscle invasive mass in trigone, treated with TURBt			Death at 14 weeks	Kramer *et al.*[5]
						2012
75 M	Intermittent hematuria	Right wall with near obliteration of ureter and invasion into fat	Glycogen positive	Prostatic adenocarcinoma	Alive at ten months	Present case
			Mucin negative	Gleason grade 3+3 (<5%)		2013
			Lipid negative			
			PSA negative			

F, female; M, male; PSA, Prostate-specific antigen; PSAP, Prostatic acid phosphatase; TURBt, transurethral resection of bladder tumor. EM, electron microscopy.

Although the presence of clear cells in otherwise typical urothelial carcinoma is not uncommon [3,4], the tumor in this case had clear cells far in excess of what is normally seen in urothelial carcinoma. To the best of our knowledge, no study to date has shown that clear cell dysplasia progresses to clear cell urothelial carcinoma [3,4]. “In fact, the infrequent reporting of clear-cell transitional cell carcinoma, when compared with the relatively common observation of clear-cell dysplasia, makes this unlikely,” per Braslis *et al.*[4].

Regarding PAS staining, Braslis and colleagues similarly reported dense and spotty positivity for cytoplasmic glycogen in one of their cases of clear cell urothelial carcinoma (Table 2) [4]. Normal urothelium contains glycogen, and positive reactivity of urothelial carcinomas for glycogen was explored by Kotliar and colleagues [6]. They selected 24 random urothelial carcinomas ranging from low-grade superficial papillary tumors to high-grade invasive tumors [6]. Approximately two thirds of tumors, despite their non-clear cytoplasm, showed varying degrees of PAS positivity that disappeared with diastase digestion (PAS-D). The overall apparent pattern of PAS/PAS-D reactivity correlated with tumor grade; in general, low-grade superficial urothelial cell carcinomas tended to have stronger diffuse staining, whereas poorly differentiated tumors tended to have negative or focal positivity [6].

### Differential diagnosis

The finding of a clear cell carcinoma in the urinary tract usually implies the diagnosis of an adenocarcinoma [6]. Clear cell adenocarcinomas show a distinct predominance affecting the female urethra, although it may occur in men and in the urinary bladder [6]. These adenocarcinomas are characterized by tubules, cysts, papillae, or diffuse sheets of clear cells containing abundant, clear glycogen-rich cytoplasm [1]. The tumor cells lining the tubules and cysts may be cuboidal, hobnail, or flattened and usually show strong immunostaining for PAX 8 [1,8]. A signet-ring cell type is observed in some cases and is distinguished by abundant intracytoplasmic mucin globules of varying size displacing the nucleus to the cell periphery [4]. By contrast, clear cell urothelial carcinomas, as observed in our case, do not show luminal formation or hobnail cells, and are mucin and PAX8 negative.

The lipoid-cell variant of urothelial carcinoma could also be considered in the differential diagnosis. De Giorgi and colleagues described a bladder tumor featuring poorly differentiated, pleomorphic cells with nuclear pleomorphism and large, optically clear intracytoplasmic vacuoles imparting an adipocytic appearance [9]. Leroy and colleagues did stains on such tumors and found that mucin stain, alcian blue and PAS stains were negative, implying lipid content [10]. By contrast, clear cell urothelial carcinomas, as observed in our case (oil red O negative), show no lipid cytoplasmic content.

Though less likely in the differential diagnoses, we could still consider a nephrogenic adenoma, especially the variant of a nephrogenic adenoma-like clear cell carcinoma. Herawi and colleagues did a study comparing clear cell adenocarcinoma, nephrogenic adenoma-like clear cell adenocarcinoma, and nephrogenic adenoma [11]. Features discriminating nephrogenic adenoma-like clear cell adenocarcinoma from nephrogenic adenoma included occasional clear cells, a prominent pleomorphism, and extensive muscular invasion [11]. The cytologic atypia of nephrogenic adenoma falls short of that seen in our case of clear cell urothelial carcinoma. In addition, nephrogenic adenoma has a low Ki-67 rate (0% to 5%) and is negative for p53, whereas in our case the Ki-67 and p53 nuclear expressions were very high (>70% and approximately 30%, respectively) [11].

The most likely clear cell metastatic disease to consider would be renal cell carcinoma. However, renal cell carcinoma metastasis to the bladder is very rare, with only approximately 30 cases reported in the literature [7]. On histology, renal cell carcinoma is frequently characterized by compact nests of cells with clear, abundant cytoplasm and delicate blood vessels [12]. This vascular network was not present in our case. In difficult cases, immunohistochemistry may be helpful. Renal cell carcinoma is typically negative for CK7 and CK20, but positive for RCC antigen, CAM 5.2, Vimentin, and PAX 8, in contrast to our case presented here [7].

Clear cell carcinomas can also arise from other organs including prostate, lung, breast, uterus, ovary, and vagina [4,5,7]. The latter sites are in females, and thus not applicable to this case. Prostatic origin however, is a consideration because our patient was discovered to have prostatic adenocarcinoma. Nevertheless, the prostate cancer observed in this case was low grade and focal (<5% of the gland was involved). As described previously, no glandular formation was seen in the bladder tumor, the clear cell vacuoles were negative for lipid content (lipid material has been reported in prostatic adenocarcinoma [4]), and the tumor was p63 positive and PSA negative.

Before identifying the tumor as a carcinoma, we also considered metastatic melanoma, clear cell sarcoma, and seminoma. These other possibilities were excluded by negative staining for S-100 protein, HMB-45, CD117, and PLAP, whereas positive staining for CK7 supported the diagnosis of carcinoma.

### Prognosis

The clinical course of clear cell variant urothelial carcinoma of the bladder is currently not known due to the lack of a larger number of cases (Table 2) [5]. Our patient in this case was alive ten months after initial presentation. In one of the cases reported by Braslis and colleagues, the longest patient survival was reported as at a six-month follow-up [4]. A less aggressive course was also reported in two patients who were treated with transurethral resection of the bladder tumor; even though one had detrusor muscle invasion, both were free from recurrence after seven and 20 months [5,7,13].

In a case report by Kramer and colleagues, the urothelial clear cell carcinoma showed a very aggressive behavior with rapid local recurrence and development of peritoneal carcinomatosis from which the patient survived only 14 weeks after diagnosis [5]. A similar aggressive course was described by Kotliar and colleagues, in which a 71-year-old man underwent a radical cystoprostatectomy and two pelvic lymph nodes were found to be positive for metastatic disease; despite adjuvant chemotherapy, their patient died after 20 months [5,6].

### Future directions

Hepatocyte nuclear factor-1β was tested in clear cell adenocarcinomas of the bladder and urethra by Brimo and colleagues in 18 cases and was found to be a useful marker in differentiating clear cell adenocarcinomas from invasive high-grade urothelial carcinoma and other types of bladder adenocarcinomas [14]. It would be interesting to apply the marker to clear cell urothelial carcinoma to learn about the staining pattern of the tumor and, possibly, for diagnostic utility.

## Conclusion

The glycogen-rich, clear cell variant of urothelial carcinoma is an exceedingly rare tumor which we report in a case where malignant clear cells were far in excess of that seen in usual urothelial carcinomas. Immunohistochemical and histochemical stains helped exclude more common malignant tumors primary to the bladder, as well as metastatic lesions. Given the small number of reported cases, prognosis is not known; the continued report of these cases may eventually shed light on the biology and prognosis of these very rare tumors. Our patient is alive at the time of writing and constantly followed in our hospital system.

## Consent

Written informed consent was obtained from our patient for publication of this case report and accompanying images. A copy of the written consent is available for review by the Editor-in-Chief of this journal.

## Abbreviations

CAM: Cell adhesion molecule; CD: Cluster of differentiation; CK: Cytokeratin; HMB: Human melanoma black; PAS: Periodic acid-Schiff; PAS-D: diastase; PAX: Paired box protein; PLAP: Placental alkaline phosphatase; PSA: Prostate-specific antigen; RCC: Renal cell carcinoma; CA: Cancer Antigen.

## Competing interests

The authors declare that they have no competing interests.

## Authors’ contributions

VMK and FGLR were the major contributors in writing the manuscript. SW performed the surgical procedure. FGLR, MSL, and VMK performed the histological examination of the renal tumor, interpreted, and diagnosed the pathology findings. VMK and WBB prepared the tables. FGLR took the microscopic pictures and edited the figures. FGLR, MSL, and WBB reviewed the manuscript. All authors read and approved the final manuscript.
